# Proof-of-Concept Double-Blind Placebo-Controlled Trial Measuring Cartilage Composition in Early Rheumatoid Arthritis under TNF-α-Inhibitor Therapy

**DOI:** 10.3390/jcm12062306

**Published:** 2023-03-16

**Authors:** Miriam Frenken, Benedikt Ostendorf, Ralph Brinks, Christoph Schleich, Lena M. Wilms, Stefan Vordenbäumen, Anja Müller-Lutz, Jutta G. Richter, Oliver Sander, Gerald Antoch, Matthias Schneider, Xenofon Baraliakos, Daniel B. Abrar, Philipp Sewerin

**Affiliations:** 1 Department of Diagnostic and Interventional Radiology, Medical Faculty, University Hospital Düsseldorf, Heinrich-Heine-University Düsseldorf, 40225 Düsseldorf, Germany; 2 Department and Hiller Research Unit of Rheumatology, Medical Faculty, University Hospital Düsseldorf, Heinrich-Heine-University Düsseldorf, 40225 Düsseldorf, Germany; 3 Rheumazentrum Ruhrgebiet, Medical Faculty, Ruhr-University Bochum, 44649 Herne, Germany

**Keywords:** rheumatoid arthritis, therapeutics, articular cartilage, radiology, musculoskeletal system

## Abstract

Low levels of delayed gadolinium-enhanced magnetic resonance imaging of cartilage (dGEMRIC) values are indicative of cartilage degeneration. Patients with early rheumatoid arthritis are known to have low dGEMRIC values due to inflammatory activity. The additional effect of biological disease-modifying antirheumatic drug (bDMARD) and conventional synthetic disease-modifying antirheumatic drug (csDMARD) treatment on cartilage status is still unclear. In this prospective, double-blinded, randomized proof-of-concept clinical trial, patients with early rheumatoid arthritis (disease duration less than 12 months from symptoms onset) were treated with methotrexate + adalimumab (10 patients: 6/4 (f/m)). A control group with methotrexate alone (four patients: 2/2 (f/m)) was used. Cartilage integrity in the metacarpophalangeal joints was compared using dGEMRIC at baseline, 12, and 24 weeks after treatment initiation. A statistically significant increase in dGEMRIC levels was found in the adalimumab group considering the results after 12 and 24 weeks of therapy (*p* < 0.05) but not in the control group (p: non-significant). After 24 weeks, a tendency towards increased dGEMRIC values under combination therapy was observed, whereas methotrexate alone showed a slight decrease without meeting the criteria of significance (dGEMRIC mean change: +85.8 ms [−156.2–+346.5 ms] vs. 30.75 ms [−273.0–+131.0 ms]; p: non-significant). After 24 weeks of treatment with a combination of methotrexate and adalimumab, a trend indicating improvement in cartilage composition is seen in patients with early rheumatoid arthritis. However, treatment with methotrexate alone showed no change in cartilage composition, as observed in dGEMRIC sequences of metacarpophalangeal joints.

## 1. Introduction

Rheumatoid arthritis (RA) is a chronic systemic autoimmune disease characterized by enduring joint inflammation resulting in a specific pattern of cartilage and bone damage and ultimately loss of joint function (1). The chronic, progressive nature of the disease leads to physical limitation, reduced quality of life, and higher mortality rates [1,2].

The pathomechanism of RA is not yet fully understood. However, inflammation of the synovial membrane, cytokine, and chemokine-induced cell migration into the joint space, and consecutive cartilage damage and bone erosions, appear to play a key role in the progression and maintenance of the disease [3].

Early detection, accurate monitoring, and a treat-to-target approach with disease-modifying antirheumatic drugs (DMARDs) are components of a modern treatment strategy that aims to control inflammation soon after diagnosis to prevent joint damage [4]. In the last two decades, the treatment of RA has been revolutionized by the development of biological disease-modifying antirheumatic drugs (bDMARDs). Adalimumab (ADA) as one of these is a TNF-α inhibitor approved for the treatment of patients who do not achieve clinical remission with conventional synthetic (cs) DMARDs (e.g., methotrexate, MTX) [5]. Beyond significant improvement in clinical activity already after 12 weeks [6], treatment with adalimumab also showed significantly less structural damage in conventional radiographs [7].

Magnetic resonance imaging (MRI) is more sensitive than conventional radiography in the detection of RA-related musculoskelettal alterations [8]. In 2003, the Outcome Measures in Rheumatology Clinical Trials (OMERACT) group introduced the RA MRI score (RAMRIS), a sum score that reflects the severity of synovitis, bone marrow edema, and erosions in the hand and wrist to determine the disease activity and to monitor therapy response [9]. Cartilage changes do not directly contribute to the score, although cartilage changes appear to be important in monitoring RA and are even more closely associated with physical impairment than bone damage [10], highlighting the importance of imaging procedures that focus on cartilage. Indirectly, however, cartilage damage was included in the RAMRIS in 2017 through joint space narrowing, which is a result of cartilage damage [11]. As joint space narrowing only detects an advanced state of cartilage damage, in which the cartilage thickness has already decreased, techniques to visualize early cartilage changes are urgently needed. Compositional MR imaging techniques are suitable for this purpose, among which gadolinium-enhanced MRI of cartilage (dGEMRIC) is the gold standard. This contrast agent-based MRI technique allows the detection of cartilage degeneration by revealing the loss of proteoglycans [12] and has the potential to predict joint space narrowing [13].

This highly effective MRI technique works by indirectly visualizing proteoglycans, indicating early cartilage changes [12]. After the application of a contrast agent, the negatively charged gadolinium diethylenetriamine pentaacetate anions (Gd-DTPA) enter the cartilage in an inverse relationship to the concentration of the negatively charged glycosaminoglycan side chains of the proteoglycans. A decrease in proteoglycan content in degenerated cartilage thus leads to an accumulation of paramagnetic gadolinium ions [14]. This increased gadolinium concentration results in an enhanced signal in T1-weighted sequences and a shortened T1 relaxation time in MR imaging, i.e., lower dGEMRIC values [15]. With dGEMRIC molecular cartilage, changes in early RA could already be detected at a time when morphological changes were not yet visible [16,17]. Nevertheless, data on cartilage composition for patients with RA under therapy are rare. Recently, a stable cartilage composition was reported with MTX therapy in patients with RA over 6 months [18]. On the other hand, the effect of TNF-α inhibitors on cartilage quality still remains unclear.

Therefore, our aim was to conduct a proof-of-concept prospective study on the effect of TNF-α inhibitor treatment on cartilage integrity in newly diagnosed RA patients based on dGEMRIC assessment of finger joints.

## 2. Materials and Methods

### 2.1. Study Population

The study was approved by the local ethics committee (approval number MO-LKP-719) and was conducted in line with the Declaration of Helsinki. Written informed consent was obtained from all participants individually. All patients were diagnosed for having RA and all had to fulfill the American College of Rheumatology (ACR)/European League Against Rheumatism (EULAR) 2010 classification criteria [19] The symptom duration was not allowed to be >12 months. Any treatment with cs- or b-DMARDs including methotrexate was not allowed prior to study inclusion. Furthermore, the use of prednisolone for the last 4 weeks before study entry was not allowed. All subjects were ≥18 years of age.

Since previous work has shown that metacarpophalangeal MCP2 and MCP3 joints are more frequently affected in RA [20], these two joints were selected for study inclusion and imaging evaluation. Hence, as an inclusion criterion, all patients had to have at least one swollen or tender MCP2 or MCP3 joint in at least one hand. Patients were assigned to receive either MTX (15 mg oral MTX once weekly) alone or MTX + ADA (40 mg every 2 weeks) in a ratio of 2:1 by central block randomization. 

### 2.2. MR Imaging

MRI of the dominantly affected hand was performed in all patients at baseline and at 12 and 24 weeks after the initiation of treatment in all patients. A 3T-MRI system (Magnetom Skyra syngo; Siemens Healthineers, Erlangen, Germany) was used. Subjects were imaged in a prone position with the hand extended over the head and the palm facing down (‘superman position’). For anatomical imaging, a coronal short tau inversion recovery (STIR) sequence of the hand and the wrist, a T1-weighted turbo spin echo (TSE) sequence, a T1-weighted VIBE, and two T1-weighted sequences with two different flip angles (8 and 26°) were acquired before injection of a contrast agent. A gadolinium-based MR contrast agent was applied intravenously (0.4 mL/kg body weight of Gd-DTPA2, Magnevist; Schering). After contrast agent injection, a coronal VIBE, a TSE, and a transversal SE-sequence with fat suppression were applied. The sequence parameters were selected according to a previous study and are adapted to the Magnetom Skyra syngo 3T-MRI [12] (Table 1). Morphological images of the hand were acquired. Additionally, the cartilage integrity of MCP2 and MCP3 joints was monitored by the evaluation of compositional MRI using dGEMRIC. To achieve high-resolution images with thin sections and a small FOV, the hand was placed in a dedicated receive-only 16-channel high-resolution hand coil (3T Tim Coil, Siemens Healthineers). According to previous studies, there was a delay of 40 min between the application of the contrast agent and the dGEMRIC sequence [21]. A three-dimensional dual-flip-angle gradient-echo sequence was used for T1 mapping [21]. Flip angles were defined at 5° and 26°. Ten sagittal slices with a thickness of 2 mm and a FoV of 90 × 53.5 mm were positioned perpendicular to the joint spaces. Motion correction was applied to the MCP joint of each patient to reduce movement-related artifacts using STROKETOOL (Frechen, Germany) before image analysis [22].

### 2.3. Image Analysis

Molecular imaging to visualize cartilage composition was performed using dGEMRIC on MCP2 and MCP3 joints. dGEMRIC is represented by the T1 map after injection of the contrast agent. Two separate regions of interest (ROI) were placed in the phalangeal and metacarpal cartilage of the MCP joints. Gradient echo sequences with a flip angle of 8° served as anatomical references for the algorithm by which the ROI were automatically placed in each cartilage zone [23]. Within these ROI, dGEMRIC values were recorded (T1 (in milliseconds)). A mean dGEMRIC value of MCP 2 and MCP 3, proximal and distal cartilage layer, respectively, was calculated per patient and per examination time point. To complement our results, morphologic imaging was assessed in the form of the RAMRIS. MR images were analyzed by consensus by two physicians trained in musculoskeletal imaging (CS, radiologist with 8 years of experience, and PS, rheumatologist with 8 years of experience) to assess subscores for synovitis, erosion, and edema in the hand and wrist.

### 2.4. Statistical Analysis

Statistical analysis was performed using R (Version 3.6.0, 64-bit). The mean, median, first and third quartile, minimum, and maximum for dGEMRIC values were calculated as descriptive statistics (listed in Table 2). To investigate whether treatment with ADA results in a difference in cartilage composition after 6 months of therapy, we performed two calculations. First, the change in dGEMRIC values in the MCP2 and MCP3 joints was calculated separately for each of the two treatment arms including all three study time points (baseline, 12 weeks, and 24 weeks after treatment initiation). Second, the Wilcoxon rank sum test was used to directly compare the difference in dGEMRIC values between baseline and 24 weeks after therapy initiation in both study arms (ADA + MTX or placebo + MTX). *p*-values below 0.05 were considered to be significant.

## 3. Results

### 3.1. Patient Population

Initially, a total of 20 patients were included as a proof-of-concept scenario (11 females/9 males, mean age: 45.8 years). All patients received at least one MRI scan. Despite reminder letters, three patients appeared only for the first MRI appointment (3 males: 1 × ADA+ MTX group, 2 × control group). Three others received two MRI examinations and did not attend the final appointment (3 females: 2 × ADA + MTX group, 1 × control group). This resulted in 14 remaining patients, 10 allocated to the ADA + MTX group (6 females/4 males, mean age: 44.9 years) and 4 to the control group (2 females/2 males, mean age: 46.7 years).

### 3.2. Descriptive Statistics

**Figure 1:**
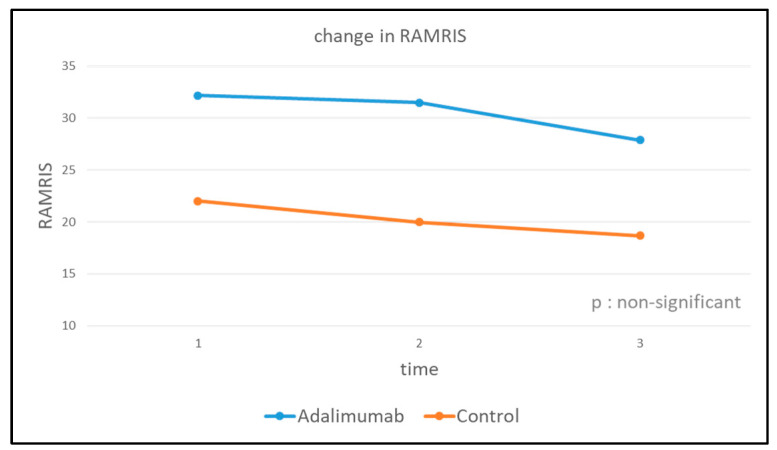
Change in RAMRIS between baseline and 24-week follow-up in both treatment arms. There was no significant difference in change between both treatment arms (*p*: non-significant).

Descriptive statistics of pooled MCP2 and MCP3 dGEMRIC values at baseline (t_0_) and the change in dGEMRIC values after 24 weeks (t_2_) compared to baseline are listed in Table 2. Furthermore, the evaluation of RAMRIS can be found in Table 3 and the decrease in RAMRIS values without significant differences in change is displayed in Figure 1 (median change over 24 weeks: ADA-MTX group: −5.8; control group: −1.0, *p*: non-significant).

### 3.3. Cartilage Integrity (dGEMRIC)

**Figure 2:**
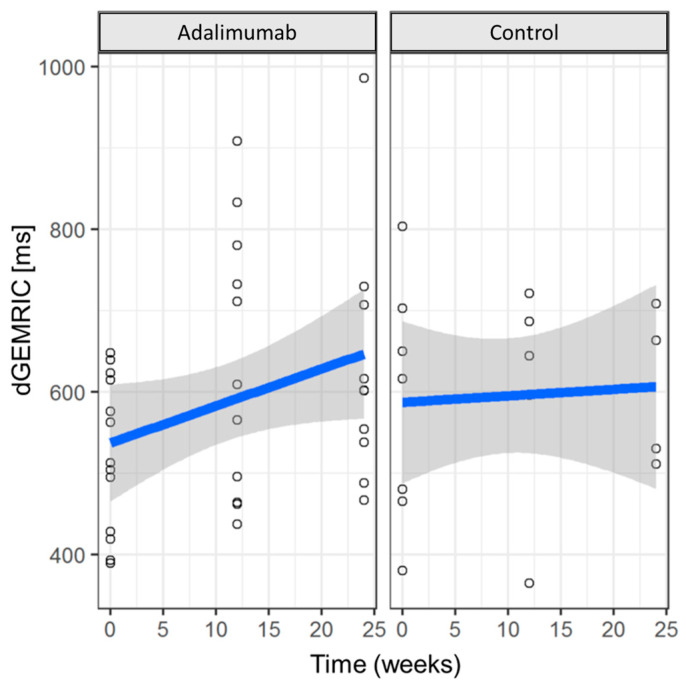
Change in cartilage quality over time. Change in dGEMRIC values in ms for both treatment groups over time with consideration of intermediate results (baseline, 12-week, and 24-week follow-up). There is strong statistical evidence of an increase in dGEMRIC values over time under adalimumab (*p* < 0.05), whereas the control group shows only a slight increase without statistical evidence (*p*: non-significant).

First, dGEMRIC values increased significantly in the ADA-MTX group (median change over 24 weeks: 75.88 ms, *p* < 0.05). In contrast, only a minimal, non-significant increase in dGEMRIC values was observed in the control group (median change over 24 weeks: 9.50 ms, p: non-significant) (Figure 2).

**Figure 3:**
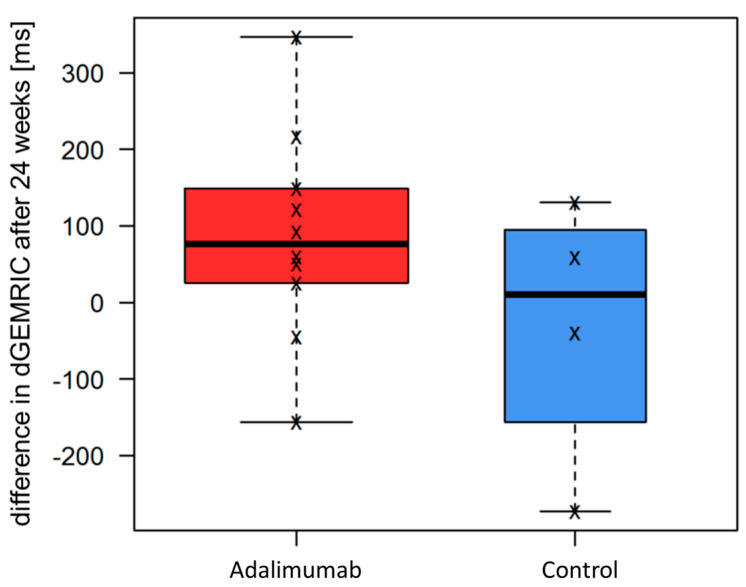
Change in cartilage quality in both treatment arms. The figure shows the difference in delayed gadolinium-enhanced MR imaging of cartilage (dGEMRIC) values in ms between baseline and 24-week follow-up in both treatment groups. Greater dGEMRIC values imply higher cartilage quality. Under adalimumab plus methotrexate (MTX), there was a—yet not significant—tendency for a greater increase in dGEMRIC values, suggesting a slight improvement in cartilage quality compared to the MTX monotherapy of the control group.

**Figure 4:**
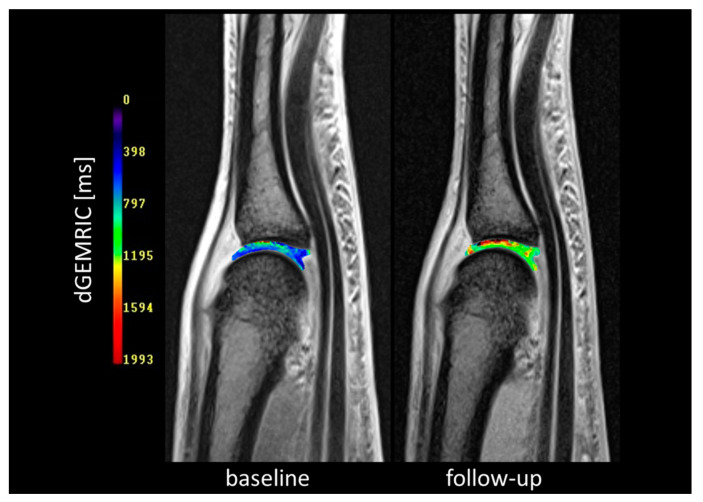
Cartilage composition of the MCP3 joint at baseline and after therapy. Fusion of morphological images with dGEMRIC maps demonstrating cartilage composition of the MCP3 joint at baseline and after 24 weeks of adalimumab plus MTX therapy (follow-up). Blue indicates cartilage damage and red indicates healthy cartilage. After therapy dGEMRIC values have increased (change from blue to green), indicating cartilage regeneration.

Second, in the direct comparison of the two treatment arms, there was a tendency for a greater increase in dGEMRIC values under adalimumab (ADA + MTX) than in the control group (placebo + MTX) which, however, did not meet the criteria for statistical significance (ADA + MTX group: dGEMRIC mean change 85.8 ms, range −156.2–346.5 ms; control group: dGEMRIC mean change −30.75 ms, range −273.0–131.0 ms; p: non-significant) (Figure 3). An example of dGEMRIC visualized cartilage of an early RA patient treated with adalimumab plus MTX is shown in Figure 4.

## 4. Discussion

An early start of a disease-modifying therapy and achieving early remission are two of the basic principles of the therapy concept for RA [4]. Early treatment significantly improves the long-term outcome and is therefore an important principle of the EULAR recommendations [4]. In this double-blind, placebo-controlled, proof-of-concept clinical trial, the effect of adalimumab in combination with MTX vs. MTX monotherapy on joint cartilage integrity of MCP2 and MCP3 in patients with early RA was examined with compositional MRI of the cartilage by using dGEMRIC [24]. To the best of our knowledge, this is the first trial evaluating the effect of adalimumab therapy on the cartilage in the finger joints.

Our main finding was that adalimumab added to MTX therapy leads to a significant increase in dGEMRIC values, which continuously improved over time, taking into account the measurements after 12 and 24 weeks, which was not observed in the group treated with MTX without adalimumab. These results indicate that controlling inflammation with adalimumab may lead to an improvement in cartilage quality, which is impaired by the underlying inflammatory disease (RA) [11]. In addition, the baseline to endpoint comparison alone, 6 months after initiation of therapy, showed a trend in cartilage improvement under ADA + MTX. However, compared with the control treatment arm, placebo + MTX, this change was not statistically relevant, possibly due to the fact that the number of patients who could be recruited was lower than expected.

The results of this study are in line with Beals et al., who documented reduced cartilage damage under bDMARD therapy using infliximab [25]. In this study, the results even suggest that, under therapy with bDMARDs, not only a reduction of cartilage damage but even a slight regeneration seems possible. The slightly different results between our study and the results of Beals et al. might be due to the different cartilage measurement MRI techniques (DCE-MRI vs. dGEMRIC) or due to the rather unlikely effect of the different bDMARDs used in both studies. Moreover, it has to be mentioned that the study populations also differ since our study aimed at very early, cs- and bDMARD naïve RA patients as compared to patients with a diagnosis of RA for at least 6 months in the study by Beals et al. However, both studies support the assumption of a positive effect of TNF-α-inhibitors on cartilage integrity in RA. Whether the effect of the TNFα inhibitor in this study can be explained predominantly by a direct influence on the cartilage matrix or by a general decrease in inflammation, which indirectly has a positive effect on the cartilage, is still unclear. In addition to compositional imaging with dGEMRIC, the RAMRIS based on morphological imaging and sensitivity to change in joint inflammation and damage has been evaluated. The decreasing RAMRIS values in both treatment groups showed no significant difference in change, suggesting that next to the decreasing inflammation, a direct effect of TNF-α -inhibitors on cartilage might be responsible for the positive compositional cartilage changes under adalimumab. The results of this study are in contrast with the only further study that investigated the effect of bDMARDs on cartilage using dGEMRIC. Tiderius et al. investigated knee joints in 7 chronic RA patients and found cartilage deterioration after 22 weeks [26]. A possible explanation is that patients with early RA respond better to bDMARDs than patients at a more advanced stage of the disease, which again supports the current treatment principle of early and treat-to-target (T2T) therapy. Another potential explanation is a difference in sensitivity to bDMARDs between different joints. This theory can be supported by findings that point out a difference in cartilage regeneration between knee and ankle joints. Kuettner and Cole showed that cartilage cells in the knee synthesize fewer proteoglycans in reaction to damage than do cartilage cells in the ankle, suggesting a lower capacity for repair and regeneration [27]. Furthermore, the results of Kuettner and Cole indicated an increased degree of cartilage surface disorder with increasing body weight. In our cohort, however, there were no significant differences with respect to body weight.

Recently, the suitability of MRI for therapy monitoring using the control parameter bone edema has been questioned, as superiority of the MRI-guided treat-to-target group compared to the conventionally guided group could not be demonstrated [28]. This study explicitly focused on cartilage quality measured by dGEMRIC, which can only be assessed by MRI. Compositional cartilage imaging is expected to detect cartilage damage at an early stage [13]. As this may be a sign of progressive RA, which should be treated as early as possible [11], we still consider MRI a valuable diagnostic tool, especially since cartilage changes might be a more sensitive control parameter than bone marrow edema.

This study has some strengths. It is a double-blinded, placebo-controlled, clinical trial and the first published report of the compositional ability of MRI to depict cartilage regeneration in RA. This is important since the exclusion of the assessment of cartilage in previous clinical trials was an obstacle to the acceptance of MRI as a substitute for radiography [29]. However, the use of MRI is preferable as previous studies have shown that cartilage loss is at least as relevant to the long-term outcome as bone erosion [29]. One of the further strengths of this study is the use of dGEMRIC for cartilage imaging, as until recently, the MRI technique dGEMRIC was considered the reference standard to evaluate extracellular matrix components of hyaline cartilage [21]. 

Limitations of this study include manually selected ROI for dGEMRIC measurement in the MCP finger joints instead of automatically algorithm-based ROI. Due to the small size of the MCP joint and the resulting low contrast, automated cartilage recognition is not yet reliable enough to be used in study situations. Manual evaluation by an experienced radiologist was therefore preferred, despite the possible human interference factor. For future studies, the increased automated evaluation of clinical image data should be pursued. Another limitation of the current study was that the observations were based on a small number of patients. Only patients with early rheumatoid arthritis, i.e., with an onset of symptoms less than 12 months ago, were included in this patient group. These patients are relatively difficult to enroll, as the path from first symptom to the rheumatological diagnosis is often time-consuming. In addition, some of these patients start therapy with DMARDs, which was an exclusion criterion for the study. Recruitment of the patients was therefore only possible through close cooperation with the local outpatient clinic and doctors from primary care. To validate the changes in finger joint cartilage measured in patients with early rheumatoid arthritis in this proof-of-concept scenario, future studies with larger patient cohorts are recommended.

## 5. Conclusions

In conclusion, this study suggests that in patients with early RA, Adalimumab plus MTX tends to improve cartilage composition after 24 weeks. The results point out that TNF-α-inhibitors may induce cartilage regeneration in early RA and thus slow down or even prevent the development of joint damage, while this is not the case for patients treated with MTX alone. Furthermore, compositional MR imaging is proving to be a valuable tool for assessing inflammation to detect early cartilage damage in RA. 

## Figures and Tables

**Table 1:** Detailed sequence parameters.

Sequence/Parameter	STIR	T1 TSE	T1 VIBE	T1 FLIP	T1 VIBE	T1 TSE	T1 TSE fs	T1
Contrast agent	no	no	no	no	yes	yes	yes	yes
Orientation	coronal	coronal	coronal	coronal	coronal	coronal	transversal	sagittal
TE/TR [ms/ms]	31/ 5560	27/ 862	5.8/ 1.9	5.8/ 1.9	5.8/ 1.9	27/ 862	16/ 702	4.56/ 15
Flip angle [°]	120	150	8	8 + 26	8	150	90	5 + 26
Slice thickness [mm]	2.5	2.5	3	3	3	2.5	2.5	2
FoV [mm × mm]	130 × 130	140 × 140	140 × 140	140 × 140	140 × 140	140 × 140	120 × 120	90 × 53.5
Number of images	1	1	1	2	1	1	1	2
Basic resolution	448	512	128	128	128	512	384	384
Number of acquired slices	17	17	10	10	10	17	20	10

STIR: short tau inversion recovery; TSE: turbo spin echo; VIBE: volumetric interpolated breath-hold examination; Fs: fat-suppressed; TE: echo time; TR: repetition time; FoV: field of view.

**Table 2:** dGEMRIC values at baseline (t_0_) and change in dGEMRIC values after 24 weeks (t_2_) compared to baseline.

	Adalimumab		Control Group	
Pooled MCP2 and MCP3 dGEMRIC Values	t_0_	Δ(t_2_ − t_0_)	t_0_	Δ(t_2_ − t_0_)
Min.	390	−156.20	380.2	−273.00
1st Qu.	428.5	31.62	473.2	−97.88
Median	513.0	75.88	616.0	9.50
Mean	523.7	85.78	585.5	−30.75
3rd Qu.	614.8	141.60	676.2	76.62
Max.	647.8	346.50	803.2	131.00

MCP2/3: metacarpophalangeal joint of digitus 2/3; dGEMRIC: delayed gadolinium-enhanced MRI of cartilage; MTX: methotrexate.

**Table 3:** RAMRIS values at baseline (t_0_), after 12 weeks (t_1_), after 24 weeks (t_2_) in both treatment groups.

RAMRIS			
Adalimumab	t_0_	t_1_	t_2_
Max.	75	70	57
Median	32.18	31.50	27.90
Min.	10	8	8
Control group			
Max.	29	28	28
Median	22.00	20.00	18.67
Min.	17	14	12

RAMRIS: rheumatoid arthritis magnetic resonance imaging score; MTX: methotrexate.

## Data Availability

Data and evaluation scripts can be provided by the authors upon reasonable request.
